# Correlation between two- and three-dimensional crystallographic lattices for epitaxial analysis. II. Experimental results

**DOI:** 10.1107/S2053273322002170

**Published:** 2022-04-11

**Authors:** Josef Simbrunner, Jari Domke, Falko Sojka, Andreas Jeindl, Felix Otto, Marco Gruenewald, Oliver T. Hofmann, Torsten Fritz, Roland Resel, Roman Forker

**Affiliations:** a Division of Neuroradiology, Vascular and Interventional Radiology, Medical University Graz, Auenbruggerplatz 9, Graz, 8036, Austria; b Institute of Solid State Physics, Friedrich Schiller University Jena, Helmholtzweg 5, Jena, 07743, Germany; c Institute of Solid State Physics, Graz University of Technology, Petersgasse 16, Graz, 8010, Austria

**Keywords:** organic epitaxy, GIXD, LEED, thin films, indexing, polymorphism

## Abstract

While the crystal structure of the polymorph phase can be studied in three dimensions conveniently by X-ray methods like grazing-incidence X-ray diffraction (GIXD), the first monolayer is only accessible by surface-sensitive methods that allow the determination of a two-dimensional lattice. Here, GIXD measurements with sample rotation are compared with distortion-corrected low-energy electron diffraction (LEED) experiments on conjugated molecules: 3,4;9,10-perylenetetra­carb­oxy­lic dianhydride (PTCDA), 6,13-pentacene­quinone (P2O), 1,2;8,9-dibenzopentacene (*trans*-DBPen) and di­cyano­vinyl-quaterthio­phene (DCV4T-Et2) grown by physical vapor deposition on Ag(111) and Cu(111) single crystals. For these molecular crystals, which exhibit different crystallographic lattices and crystal orientations as well as epitaxial properties, the geometric parameters of the three-dimensional lattice are compared with the corresponding geometry of the first monolayer. A comparison of the monolayer lattice from LEED investigations with the multilayer lattices determined by rotated GIXD experiments reveals a correlation between the first monolayer and the epitaxial growth of three-dimensional crystals together with lattice distortions and re-alignment of molecules. The selected examples show three possible scenarios of crystal growth on top of an ordered monolayer: (i) growth of a single polymorph, (ii) growth of three different polymorphs; in both cases the first monolayer serves as template. In the third case (iii) strong lattice distortion and distinct molecular re-alignments from the monolayer to epitaxially grown crystals are observed. This is the second part of our work concerning the correlation between two- and three-dimensional crystallographic lattices for epitaxial analysis. In the first part, the theoretical basis has been derived which provides a mathematical relationship between the six lattice parameters of the three-dimensional case and the three parameters obtained for the two-dimensional surface unit cell, together with their orientation to the single-crystalline substrate. In this work, a combined experimental approach of GIXD and LEED is introduced which can be used to investigate the effect of the epitaxial monolayer on the structural properties of molecular crystals grown on top.

## Abstract 2

Organic epitaxy is studied experimentally in terms of the correlation of the involved two- and three-dimensional crystallographic lattices.

## 1. Introduction

For an epitaxial analysis, it is desirable to determine the crystallographic lattices in the contact layer (*i.e*. first monolayer) and in the multilayer, in order to elucidate the template effect of the first monolayer on the growth of further molecular layers deposited on top (Kilian *et al.*, 2004 ▸; Wagner *et al.*, 2004 ▸; Kröger *et al.*, 2016 ▸). Analytical methods for the epitaxially grown crystals, such as rotated grazing-incidence X-ray diffraction (GIXD) (Marra *et al.*, 1979 ▸; Als-Nielsen *et al.*, 1994 ▸; Kaganer *et al.*, 1999 ▸; Nakayama *et al.*, 2016 ▸), provide information on the periodicity of a three-dimensional lattice. The monolayer, however, is only accessible in two dimensions, where distortion-corrected low-energy electron diffraction (LEED) is the method of choice (Sojka *et al.*, 2013*a* ▸,*b* ▸). The surface sensitivity of LEED is caused by the small mean free path of electrons in solids, being in the order of 10 Å or less for kinetic energies between ten and a few hundred eV (Seah & Dench, 1979 ▸; Graber *et al.*, 2011 ▸). This means that the elastic electron scattering occurs predominantly at the surface layer and only a few atomic (or molecular) layers underneath. For X-rays, on the other hand, the mean free path is considerably longer, which means that X-ray diffraction usually contains information of a much larger scattering volume and is therefore much less surface sensitive than electron diffraction. A typical problem stems from the missing link between the monolayer and multilayer thickness regimes of the investigated adsorbates when only one method (for instance, either LEED or GIXD) is employed. In general, the energetic conditions for the heteroepitaxial adsorption of atoms or molecules depend not only on the intralayer energies but on the distance from the interface with the substrate as well, and thus one expects the microscopic adsorbate structure to be a function of the film thickness. This shall be investigated here by combining GIXD and LEED measurements.

**Figure 1:**
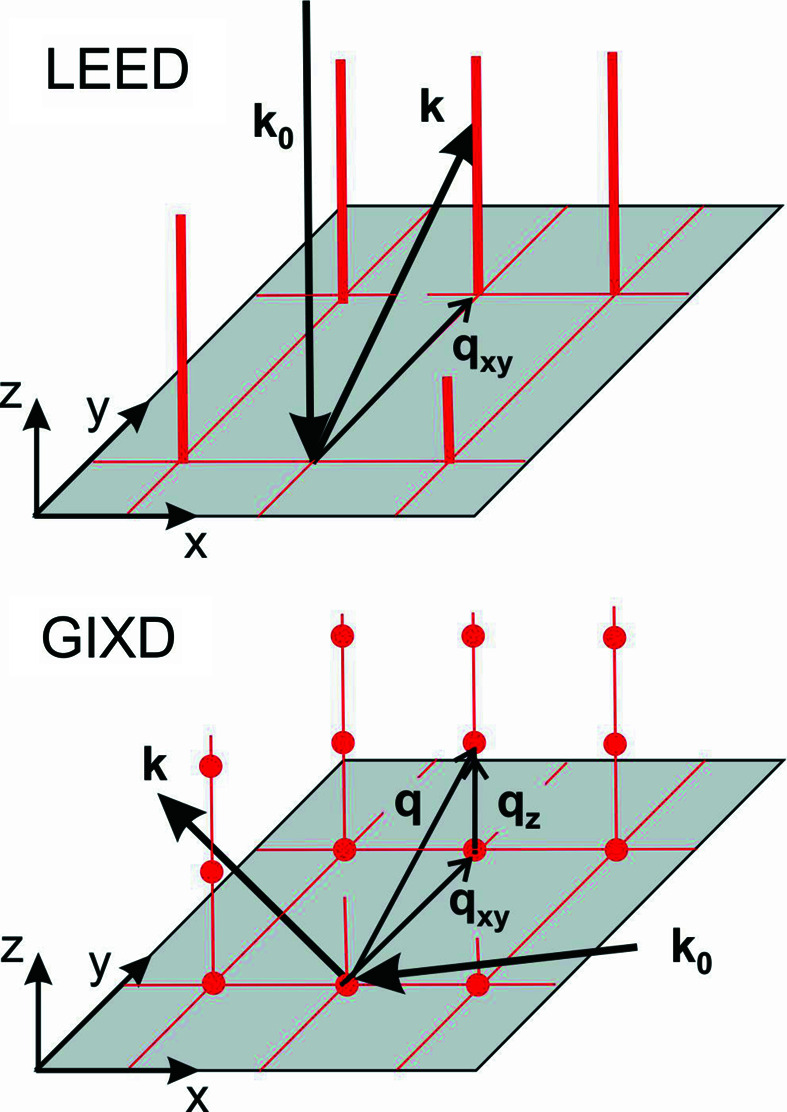
Scattering geometries of a GIXD and a LEED experiment with **k**_0_ and **k** as the wavevector of the primary and scattered radiation, respectively. In GIXD the complete scattering vector **q** is obtained experimentally, which can be split into an in-plane part **q**_*xy*_ and an out-of-plane part **q**_*z*_. In LEED only **q**_*xy*_ can be determined. The investigated reciprocal lattices are plotted in red: discrete reciprocal-lattice points in the case of GIXD and Bragg rods in the case of LEED.

In Fig. 1 ▸, the scattering geometries of a GIXD and a LEED experiment are schematically depicted. In GIXD the complete scattering vector **q** is obtained experimentally, which can be split into an in-plane part **q**_*xy*_ and an out-of-plane part **q**_*z*_. In LEED only **q**_*xy*_ can be determined. Thus, in GIXD, Bragg points can be obtained, whereas in LEED columns of Bragg points, *i.e*. lattice rods or Bragg rods, result (Robinson & Tweet, 1992 ▸).

Nowadays, GIXD experiments are performed with two-dimensional X-ray detectors which provide increased measurement efficiency. However, a reduced resolution of the Bragg peaks is obtained in comparison with classical techniques which use highly collimated diffracted beams (Smilgies, 2009 ▸; Fumagalli *et al.*, 2012 ▸). A more recent development is the rotation of the substrate during the GIXD measurement (rotated GIXD) which allows large volumes of the reciprocal space to be covered (Schrode *et al.*, 2019 ▸). Frequently, a combination of specular X-ray diffraction with GIXD is used for the characterization of thin films, since these two techniques cover different areas (or volumes) of the reciprocal space (Kowarik *et al.*, 2006 ▸). Within this work we present a combination of LEED with rotated GIXD which is an enhancement of the previously used combination of LEED with an X-ray diffraction pole figure technique (Müllegger *et al.*, 2003 ▸; Winter *et al.*, 2004 ▸).

In previous works, we performed rotated GIXD experiments on the conjugated molecules 6,13-pentacene­quinone (P2O), 3,4;9,10-perylenetetra­carb­oxy­lic dianhydride (PTCDA), 1,2;8,9-dibenzopentacene (*trans*-DBPen) and di­cyano­vinyl-quaterthio­phene (DCV4T-Et2) grown by physical vapor deposition on (quasi-)hexagonal single-crystalline surfaces like Ag(111) and Cu(111) (Simbrunner *et al.*, 2020 ▸, 2021*a* ▸). Two general crystallographic features of epitaxially grown films could be observed: (i) the crystallites grow with defined crystallographic planes parallel to the substrate surface (*i.e.* contact planes). In all our test cases, we found positive and negative orientations of the contact planes, *i.e*. the planes with the Miller indices (*uvw*) and 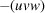 . (ii) The crystallites show additionally distinct azimuthal alignments in the *xy* plane. When Ag(111), Cu(111) or graphene/SiC(0001) were used as substrates, for each contact plane two groups of 60° symmetry were observed, one for the positive (*uvw*) and one for the negative 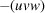 orientation, aligned symmetrically around the main axes of the substrate.

The surface unit cell is spanned by two vectors, which are linear combinations of the three vectors of the three-dimensional lattice. In the first part of our work (Simbrunner *et al.*, 2022 ▸), a comprehensive mathematical framework has been developed to correlate the parameters of the corresponding three- and two-dimensional lattices. The knowledge of the orientation and parameters of the three-dimensional crystal lattice (or unit cell) allows the interpretation of the two-dimensional data by a direct comparison of the lattices. Depending upon the Miller indices of the contact plane, either basis vectors of the three-dimensional unit cell or composites (diagonals) of them build up the corresponding surface unit cell (rhomboid). The derived mathematical formulas have been applied to previously obtained GIXD data (Simbrunner *et al.*, 2020 ▸, 2021*a* ▸). We could demonstrate that in two dimensions the positive and negative orientations of the contact planes, *i.e*. the planes with the Miller indices (*uvw*) and 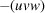 , correspond to surface unit cells with mirror symmetry about an axis along the lattice vector **a** (γ→ −γ). Thus, rotational and mirror symmetries coexist.

In this work, we will check our theoretically derived results with experimental data by indexing only *x* and *y* components of the scattering vector (*q_x_*, *q_y_*) from GIXD experiments. Mathematical expressions were derived for an indexing method (Simbrunner *et al.*, 2021*b* ▸, 2022 ▸), which will be described and proposed for this purpose. Afterwards, we will also compare these findings with the results of recent LEED experiments on the same molecules to compare the crystallographic properties of the monolayer and multilayer.

This approach reveals information about the crystal growth beyond the first monolayer (or contact layer) at the substrate surface.

## 2. Method

### 2.1. Indexing

The mathematical basis for our indexing procedure was derived in our theoretical work (Simbrunner *et al.*, 2022 ▸). A more detailed treatise was published recently (Simbrunner *et al.*, 2021*b* ▸). Please note that all vectors throughout Section 2 are entirely two-dimensional.

In our indexing procedure, pairs of reciprocal vectors in all possible combinations are formed, *i.e.* if *n* vectors are given, these are 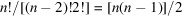 pairs {**g**_1_, **g**_2_}, where **g**_1_ and **g**_2_ are any two reciprocal vectors. The selected pairs of reciprocal vectors are combined to matrices **G** = (**g**_1_, **g**_2_). If they belong to the same system, their inverse matrices multiplied with the vectors of the corresponding Laue indices will result in the vectors of the rhomboid. This can be achieved by multiplying the inverse matrices **G** ^−1^ with vectors 2π(*m*_1_, *m*_2_)^T^, where the *m_i_* are systematically varied. Then, lattice vectors of the reduced rhomboid or of its superlattices are obtained. The vectors are sorted according to their lengths, and in ascending order, two vectors, which are not collinear, are chosen. Boundary conditions, *e.g.* for the expected vector lengths, can be used to restrict the possible solutions.

The tentative cell matrices are multiplied with all reciprocal vectors. If the scalar products yield integers (*i.e.* the corresponding Laue indices), the matrices and reciprocal vectors belong to the same system. For a system of reciprocal vectors, the rhomboid with the smallest deviations from integers will be chosen. Solutions with a larger number of associated reciprocal vectors will be preferred.

From the cell matrix, the cell parameters *a*, *b*, γ (*i.e*. the angle between **a** and **b**) and ϕ (*i.e.* the angle between **a** and the *x* axis of the laboratory system) can be obtained. Finally, the matrix of the unit-cell vectors can be optimized using various procedures.

### 2.2. Including multiple scattering in the geometric description of LEED

Multiple scattering is a common phenomenon encountered in LEED measurements (Van Hove *et al.*, 1986 ▸; Van Hove, 1991 ▸). If the reciprocal vector **g** has components of both the adsorbate and the substrate, it can be written as follows:

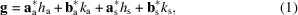

where 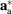 , 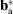 and 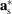 , 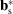 are the reciprocal-lattice vectors, and *h*_a_, *k*_a_ and *h*_s_, *k*_s_ are the Laue indices of the adsorbate and the substrate, respectively. As the reciprocal-lattice vectors of the substrate can be determined independently, in a ‘first guess’ the reciprocal vectors of the adsorbate are determined assuming scattering of zeroth order, *i.e*. *h*_s_ = *k*_s_ = 0. At room temperature, the lattice parameters are *a*_s_ = *b*_s_ = 2.889 Å for Ag(111) (Guo *et al.*, 2016 ▸) and *a*_s_ = *b*_s_ = 2.556 Å for Cu(111) (Lu & Chen, 2009 ▸); in both cases γ_s_ = 120° (sometimes, 60° is used instead). Then the Laue indices are chosen such that the residual error for each measured reciprocal vector **q** is as small as possible. The order of multiple scattering is determined by the highest absolute value of the Laue indices *h*_s_ and/or *k*_s_.

### 2.3. Epitaxy matrix

A two-dimensional matrix can be used that describes the epitaxial interface (Forker *et al.*, 2017 ▸). For the epitaxy matrix **M**,

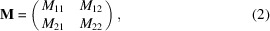

the following relation is valid:

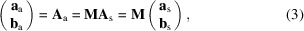

where **a**_a_ and **b**_a_ are the lattice vectors of the adsorbate (molecular contact layer), **a**_s_ and **b**_s_ are the lattice vectors of the substrate, and **A**_a_ and **A**_s_ are the associated matrices. As 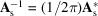 (see Simbrunner *et al.*, 2021*b* ▸), the following relation can be deduced:

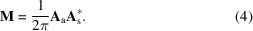

For the determinant of **M**, the following relation is valid:

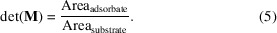

In the special case of commensurism (‘point-on-point’), all elements of the matrix **M** are integers. Therefore, each lattice vector of the adsorbate is a linear combination of the substrate lattice vectors with integer coefficients. However, other types of epitaxial registries are well known to occur frequently, especially on-line coincidences (Kilian *et al.*, 2004 ▸; Kasemann *et al.*, 2009 ▸; Kröger *et al.*, 2010 ▸, 2016 ▸; Kleimann *et al.*, 2014 ▸; Dreher *et al.*, 2020 ▸). For a review and classification see Forker *et al.* (2017 ▸).

## 3. Experimental details

### 3.1. Molecules and sample preparation

Organic semiconductors were selected for this study. Well known molecules were chosen, such as PTCDA (CAS No. 128-69-8) and P2O (CAS No. 3029-32-1), but also less well characterized molecules like *trans*-DBPen (CAS No. 227-09-8) and side-chain-substituted DCV4T-Et2 (CAS No. 1449383-43-0). The molecules have predominantly rigid character, but DCV4T-Et2 (obtained in sublimed form from Heliatek GmbH) shows some flexibility in the molecular conformation. P2O (Sigma Aldrich), PTCDA (Sigma Aldrich) and *trans*-DBPen (TCI Deutschland GmbH) were purchased and purified by temperature-gradient vacuum sublimation using a DSU-05 (CreaPhys GmbH) sublimation unit prior to use. All depositions were carried out in ultra high vacuum (UHV) environments with a base pressure lower than 5 × 10^−8^ Pa. Molecules were evaporated from shutter-controlled effusion cells held at a constant temperature with the samples kept at room temperature. Monolayers (typical thicknesses of ∼0.3 nm) were deposited for the LEED studies. The film growth was monitored *in situ* using differential reflectance spectroscopy (DRS) (Forker & Fritz, 2009 ▸; Forker *et al.*, 2012 ▸), stopping the deposition process as soon as a clear monolayer signal became apparent when monolayers were desired. For the PTCDA samples, excess molecules above one monolayer were removed by careful annealing of the sample until only the most strongly bound first molecular layer remained on the surface as indicated by DRS. The nominal thicknesses of the films used in the GIXD experiments were in the range from 10 to 30 nm, as estimated from the optically monitored growth and by assuming a constant molecular flux. For *trans*-DBPen this was supported by means of a quartz crystal microbalance. Single metal crystals were purchased from MaTecK GmbH. The silver single crystal was prepared by repeated cycles of Ar^+^ sputtering at 700 eV with incident angles of ±45° relative to the surface normal and subsequent annealing at 770 K; for copper an incident angle of 60° was used instead and the crystal was rotated during sputtering. A sufficient surface quality was confirmed by LEED before deposition of the molecular films.

### 3.2. GIXD

The experimental details were given previously (Simbrunner *et al.*, 2021*a* ▸). GIXD measurements were performed at the XRD1 beamline, synchrotron Elettra, Trieste, Italy, using a wavelength of 1.4000 Å and a stationary Pilatus 2M detector. Samples were rotated around their surface normal during the GIXD measurements. Extraction of peak positions was performed manually as already described in the literature (Simbrunner *et al.*, 2020 ▸). Due to the large detector, data from the left-hand side (LHS) and the right-hand side (RHS) of the reciprocal-space map [−*q_xy_* and +*q_xy_*, respectively, *q_xy_* = (*q_x_* ^2^ + *q_y_* ^2^)^1/2^] could be evaluated. The information of a single detector side, however, is sufficient for a complete monitoring of the accessible reciprocal space. In our previous studies, we have shown that there is no significant difference in the results obtained from the RHS and LHS (Simbrunner *et al.*, 2020 ▸, 2021*a* ▸). Therefore, in this study, we included only the data from the RHS.

### 3.3. LEED

LEED experiments were carried out using two separate microchannel-plate LEED devices ‘BDL800IR MCP2’ (OCI Vacuum Microengineering, Inc.). These devices operate at a very low primary electron flux which drastically reduces the probability of beam-induced damage. All images were corrected for distortions using the software *LEEDCal* (Sojka & Fritz, 2021*a* ▸). Spot positions in reciprocal space were extracted using the software *LEEDLab* (Sojka & Fritz, 2021*b* ▸). Lattice simulations were performed using the software *LEEDLab* (Sojka & Fritz, 2021*b* ▸), based on geometric LEED theory.

### 3.4. Scanning tunneling microscopy (STM)

For STM measurements a JT-STM/AFM (SPECS Surface Nano Analysis GmbH), equipped with an Ar^+^-sputtered tungsten tip, was used and operated at 4.5 K. Data analysis was performed with the open-source software *Gwyddion* (Nečas & Klapetek, 2012 ▸).

## 4. Results and discussion

### 4.1. General remarks

The selected molecules, together with their single-crystalline substrates, represent a variety of systems for epitaxial growth with defined molecule/substrate combinations. For the three-dimensional unit cell, we used our results on the conjugated interfaces we had studied in previous rotated GIXD experiments, *i.e*. P2O/Ag(111), PTCDA/Ag(111), DCV4T-Et2/Ag(111) and *trans*-DBPen/Cu(111) (Simbrunner *et al.*, 2020 ▸, 2021*a* ▸). Indexing in two dimensions was performed for the *x* and *y* components of the reciprocal-space vectors (*q_x_*, *q_y_*) of these GIXD data, and for the partly unpublished recent LEED measurements being discussed here. The studies on PTCDA/Ag(111) and on P2O/Ag(111) have been published very recently (Simbrunner *et al.*, 2021*b* ▸). In the first part of our work (Simbrunner *et al.*, 2022 ▸), we derived the mathematical framework to calculate the parameters of the two-dimensional lattice from the parameters of the associated three-dimensional lattice. In this work, we prove our results experimentally and compare these data with our recent data from LEED studies on the same molecules and substrates to correlate the properties of the multilayer with those of the monolayer.

For the area of the two-dimensional unit cell, the following relation is valid (Simbrunner *et al.*, 2022 ▸):

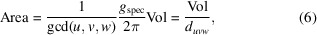

where gcd(*u*, *v*, *w*) is the greatest common divisor of the Miller indices *u*, *v* and *w*, *g*_spec_ is the length of the scattering vector of the specular diffraction peak, *d_uvw_* is the interplanar distance and Vol is the volume of the three-dimensional unit cell. We will compare the predicted values with the experimentally obtained results for our examples.

Keeping the notation in the theoretical part of our work, the components of the two-dimensional unit cells will be indicated by a prime.

### 4.2. PTCDA on Ag(111)

**Figure 2:**
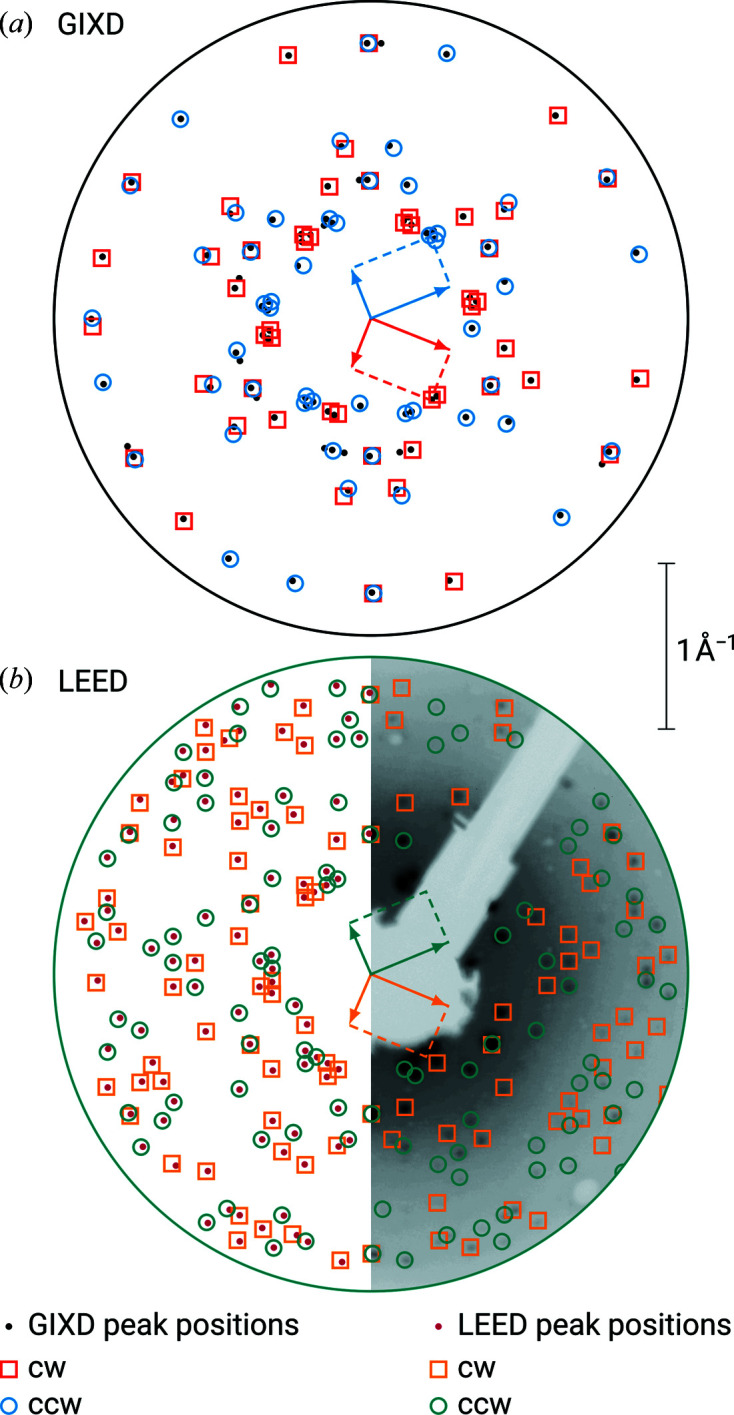
Experimentally determined (*q_x_*, *q_y_*) positions of the diffraction peaks of PTCDA crystals (small filled circles) epitaxially grown on Ag(111), obtained from (*a*) rotated GIXD and (*b*) LEED measurements. The outer circles in both panels are a guide to the eye indicating the field of view; the center in each case is the origin of the (*q_x_*, *q_y_*) coordinate system. The scale bar applies equally to both panels. Open symbols represent the results of the indexing of the oriented crystals, rotated clockwise (cw, red or orange squares) and counter-clockwise (ccw, blue or teal circles) with respect to the 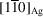 direction. Representative reciprocal unit cells for one cw and one ccw rotation are drawn in both panels. Note that the indexing results for both groups of azimuthal alignments, each with a 60° symmetry, are illustrated. Furthermore, half a LEED image is depicted to scale in the background of panel (*b*).

The epitaxy of thin films of PTCDA grown on Ag(111) has been studied using various methods, including GIXD and LEED (Glöckler *et al.*, 1998 ▸; Krause *et al.*, 2001 ▸, 2002 ▸; Kilian *et al.*, 2004 ▸; Tautz, 2007 ▸). In our studies, 180 pairs of (*q_x_*, *q_y_*) were included from the GIXD experiment and 200 reciprocal-lattice vectors were obtained from the LEED measurement. For both the GIXD as well as the LEED data sets, the indexing procedure resulted in 12 solutions with individual lattice vectors **a**′ and **b**′, from which the lattice constants could be determined. As previously described, two groups of azimuthal alignments, each with a 60° symmetry, were found (see Table 1 ▸). In Fig. 2 ▸(*a*), the (*q_x_*, *q_y_*) positions of the extracted diffraction peaks and the corresponding calculated values from the indexing result are shown for GIXD. In the three-dimensional GIXD experiment, these groups could be explained by the two contact planes (103) and (103). The orientation of the contact plane is usually indicated as (102); for the reason of crystallographic convention, however, it is in the monoclinic system with the supplementary angle β > 90° (103) (Simbrunner *et al.*, 2021*a* ▸). As in the particular case of PTCDA the conditions *v* = 0 and α = γ = 90° are fulfilled, the lattice vectors **a**, **b**, **c** for the contact planes (103) and (103) are collinear (**a** → −**a**, **b** → **b**, **c** → −**c**); therefore, an unambiguous assignment of the rotation angles φ to either one of those contact planes is not possible.

In two dimensions, these groups of azimuthal angles belong to two surface unit cells (rhomboids) with mirror symmetry. The LEED data demonstrate a commensurate epitaxial relationship between monolayer and substrate (see Table 2 ▸). Therefore, every diffraction point can be explained by the adsorbate alone. In Fig. 2 ▸(*b*), the (*q_x_*, *q_y_*) positions of the extracted diffraction peaks and the corresponding calculated values from the indexing result are shown for LEED.

As we have shown (Simbrunner *et al.*, 2022 ▸), for a monoclinic lattice (α = γ = 90°) γ′ equals 90°. In the LEED experiment, however, the angle γ′ is about 89°; therefore the monolayer unit cell is not rectangular, but a rectangular unit cell is indeed observed in the multilayer (see Table 1 ▸). The length of **a**′ (which corresponds to **b** in the three-dimensional lattice) is significantly shorter in the multilayer. However, there is almost no difference in the areas of the two-dimensional unit cells between the molecular monolayer and the multilayer (see Table 3 ▸). Furthermore, there is a good correlation between the predicted value from equation (6) and the experimentally obtained result.

In a previous LEED study on PTCDA/Ag(111), Kilian *et al.* found for the monolayer and second layer a unit cell with the parameters *a* = 12.61, *b* = 18.96 Å and γ = 89° and a commensurate epitaxial relationship (Kilian *et al.*, 2004 ▸). For the multilayer at 400 K they obtained a unit cell with the parameters *a* = 11.96, *b* = 19.91 Å and γ = 90° (area = 238.1 Å^2^) and two different epitaxial relationships which can both be classified as line-on-line coincident. Their data clearly match our results.

### 4.3. P2O on Ag(111)

**Figure 3:**
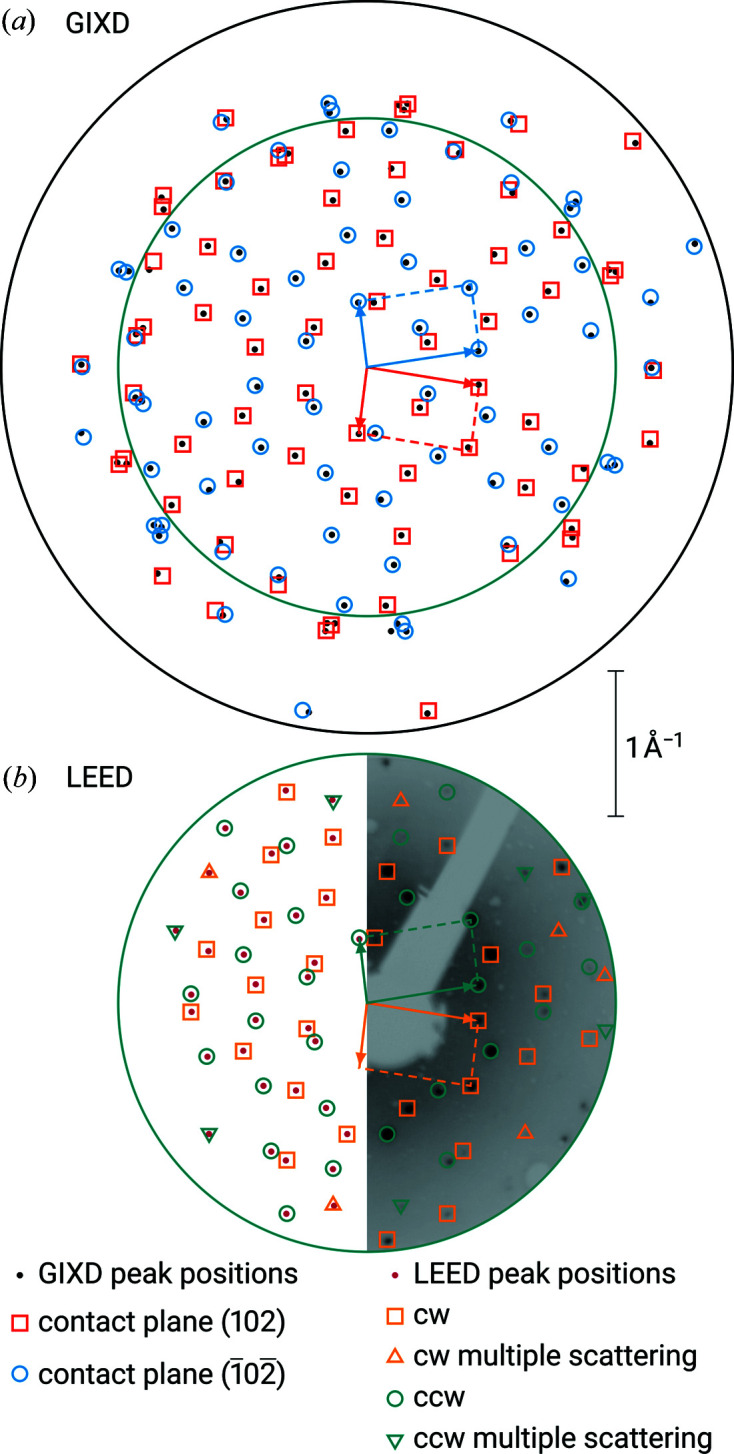
Same as Fig. 2 ▸, but for P2O on Ag(111). The second largest circle in panel (*a*) refers to the field of view of panel (*b*). Multiple scattering in the LEED experiment is differentiated by contributions of the 0th (open squares and circles) and the 1st order (upward and downward triangles), respectively.

A total of 226 pairs of (*q_x_*, *q_y_*) were included from the GIXD experiment and 70 reciprocal-lattice vectors could be obtained in the LEED measurement. In both cases, the indexing procedure on these data resulted in 12 solutions with individual lattice vectors **a**′ and **b**′, and two groups of azimuthal alignments, each with a 60° symmetry. These groups can be clearly assigned to the azimuthal alignments, which were found in the three-dimensional GIXD experiment, corresponding to the two contact planes (102) and (102). In two dimensions, these groups of azimuthal angles belong to two unit cells (rhomboids) with mirror symmetry. The group which corresponds to the (102) contact plane is rotated by about −8.7° (*i.e*. clockwise), and the group which corresponds to the (102) contact plane is rotated by +8.7° (*i.e*. counter-clockwise) with respect to the 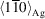 direction (see Table 1 ▸). In Fig. 3 ▸(*a*), the (*q_x_*, *q_y_*) positions of the extracted diffraction peaks and the corresponding calculated values from the indexing result are shown.

The LEED data demonstrate a point-on-line epitaxial relationship between monolayer and substrate (see Table 2 ▸). Some of the diffraction points can only be explained by multiple scattering. In Fig. 3 ▸(*b*), the (*q_x_*, *q_y_*) positions of the extracted diffraction peaks and the corresponding calculated values from the indexing result are shown, itemized for multiple scattering of zeroth and higher order and the different azimuthal alignments. Note the clear congruencies with the diffraction pattern of the GIXD experiment [Fig. 3 ▸(*a*)].

The length of **b** in the multilayer is slightly smaller than the corresponding **a**′ in the molecular contact layer (see Table 1 ▸). The area of the two-dimensional unit cells is also slightly smaller in the multilayer than in the monolayer (see Table 3 ▸).

### 4.4. DCV4T-Et2 on Ag(111)

**Figure 4:**
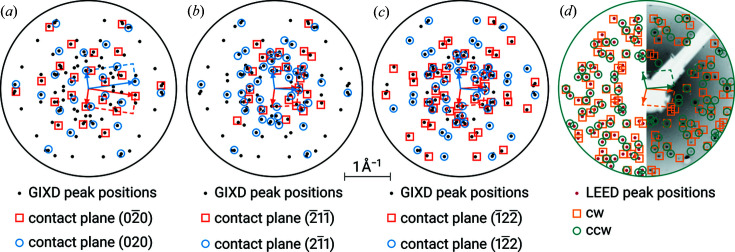
Same as Fig. 2 ▸, but for DCV4T-Et2 on Ag(111). For the sake of clarity, the GIXD results are split up into three panels, illustrating the contributions from epitaxially oriented crystals with (*a*) the 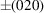 contact planes, (*b*) the 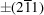 contact planes, and (*c*) the 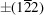 contact planes. The LEED results are depicted in (*d*).

A total of 253 pairs of (*q_x_*, *q_y_*) were included from the GIXD experiment and 171 reciprocal-lattice vectors were obtained in the LEED measurement. Indexing the LEED data resulted in 12 solutions with individual lattice vectors **a**′ and **b**′, and two groups of azimuthal alignments, each with a 60° symmetry, belonging to two unit cells with mirror symmetry. Two parameter sets with identical areas (180.6 Å^2^) could be found: *a*′ = 10.413, *b*′ = 17.567 Å, γ′ = ±80.82° and *a*′ = 10.413, *b*′ = 19.110 Å, γ′ = ±114.79°; the first set, however, contains the shorter vector **b**′ and, therefore, represents the two-dimensional Buerger cell (Buerger, 1957 ▸). The epitaxy matrix of the obtained parameters shows a clear commensurism (see Table 2 ▸). Therefore, the diffraction pattern can already be explained by the adsorbate alone. In Fig. 4 ▸(*d*), the (*q_x_*, *q_y_*) positions of the extracted diffraction peaks and the corresponding calculated values from the indexing result are shown.

In the rotated GIXD experiment performed previously, we found three polymorphs with the contact planes ±(122), ±(211) and ±(020). As explained in the first part of our work, taking our data from the rotated GIXD experiment, for the unit cell with ±(122) orientation, the following parameters could be calculated: (i) *a*′ = 11.910, *b*′ = 16.831 Å, γ′ = 78.01° and (ii) *a*′ = 11.910, *b*′ = 18.497 Å, γ′ = 117.12°. For both solutions we obtained: Area = 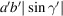 = 196.1 Å^2^. There is a clear congruency with the LEED data above. Again, solution (i) is the reduced Buerger cell. As in the previous cases, two groups of azimuthal alignments, each with a 60° symmetry, were found (see Table 1 ▸). Using equation (6) and taking our data for the volume and specular scan from our previous study, the area of the two-dimensional unit cell can be calculated to yield 195.8 Å^2^. This is in good correspondence with the number given (see Table 3 ▸). For the polymorph with ±(211) orientation, indexing the GIXD data in two dimensions gave only one solution (Simbrunner *et al.*, 2022 ▸). The cell parameters are listed in Tables 1 ▸ and 3 ▸. This cell, although slightly smaller, clearly resembles the rhomboid of the polymorph with ±(122) orientation.

Since for both contact planes none of the Miller indices is zero, no basis vector of the three-dimensional unit cell can be directly observed in the two-dimensional lattice; however, we can extract three diagonals of the parallelepiped, which are spanned by different vectors [*cf*. equations (26)–(28) in Simbrunner *et al.*, 2022 ▸]. In Table 4 ▸, we summarize the results of this analysis. This shows that there is a clear relationship between the two lattices.

The parameters of the unit cell in the ±(020) orientation are shown in Tables 1 ▸ and 3 ▸. There is some relationship between 2*a* and *c* with the corresponding parameters *a*′ and *b*′ of the other two unit cells of DCV4T-Et2/Ag(111). Furthermore, in the *xy* plane, these three polymorphs form two groups of related azimuthal alignments, each with a 60° symmetry and corresponding to the respective positive and negative contact planes (see Table 1 ▸).

It can be concluded that the three polymorphs, which can be observed in the multilayer, develop from one crystallographic lattice in the contact layer (‘parent cell’). In the multilayer the ±(122) orientation is dominant. When epitaxial graphene on silicon carbide [G/SiC(0001)] was used as the substrate, only the ±(122) orientation was observed (Simbrunner *et al.*, 2021*a* ▸). In Figs. 4 ▸(*a*)–4 ▸(*c*), the (*q_x_*, *q_y_*) positions of the extracted diffraction peaks and the corresponding calculated values from the indexing result, itemized for the different orientations and azimuthal alignments, are shown for the GIXD experiment.

### 4.5. *trans*-DBPen on Cu(111)

**Figure 5:**
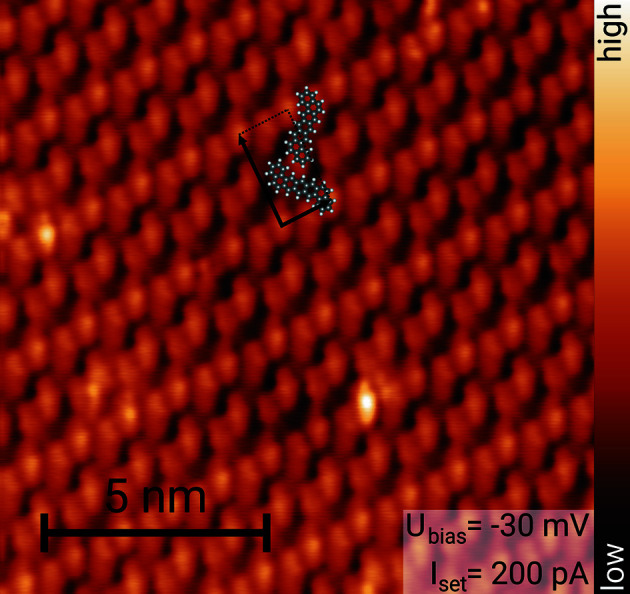
STM image of *trans*-DBPen on Cu(111) acquired at 4.5 K. The suggested structure of the unit cell containing two molecules is displayed along with the unit-cell vectors.

A total of 275 pairs of (*q_x_*, *q_y_*) were included from the GIXD measurement and 232 reciprocal-lattice vectors were obtained in the LEED experiment. Imaging by STM showed two *trans*-DBPen molecules on the surface per unit cell (Fig. 5 ▸). Hence, we searched for a unit cell with an area of at least 140 Å^2^, as this value was also obtained for *trans*-DBPen on Ag(111) with one molecule per unit cell (Otto *et al.*, 2018 ▸). We found a system of lattice vectors **a**′ and **b**′, arranged in a rectangular shape, each related azimuthal alignment exhibiting a 60° symmetry, which span an area of 306 Å^2^ (see Table 3 ▸). Angles of ±30° between **a**′ and the main axes of Cu(111) could be observed. Thus, all reflections can also be explained by a unit cell with mirror symmetry and coincidental azimuthal orientations (see Table 1 ▸). The resulting epitaxy matrix not only shows unambiguously commensurism (see Table 2 ▸), but, due to the particular values of the involved angles (γ′ of the substrate, γ′ of the adsorbate and Δϕ between the adsorbate and the substrate), one obtains for the sides of the adsorbate: 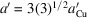 and 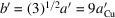 , where 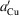 = 2.556 Å [note that the lengths of the long and short diagonals of the rhomboid spanned by Cu(111) are 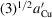 and 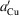 , respectively]. Thus, the substrate Cu(111) as a template exerts an especially strong influence on the contact layer.

In a previous GIXD study, we found a unit cell with the parameters *a* = 6.751 (8), *b* = 7.566 (4), *c* = 18.529 (41) Å, α = 89.88 (8), β = 86.71 (25) and γ = 89.84 (12)° and the contact planes (020) and (020) (Simbrunner *et al.*, 2021*a* ▸). For this orientation, the vectors **a**′ and **b**′ in the two-dimensional lattice correspond to the vectors **a** and **c** of the three-dimensional unit cell. Using first-principles density functional theory (DFT) with van der Waals correction, the following monoclinic unit cell for *trans*-DBPen was found: *a* = 6.745, *b* = 7.613, *c* = 18.495 Å, β = 97.13° and volume *V* = 942.5 Å^3^ (Zhong *et al.*, 2017 ▸). In our molecular dynamics (MD) simulations the best match was achieved for a herringbone structure (herringbone angle 23.3°) (Simbrunner *et al.*, 2021*a* ▸).

**Figure 6:**
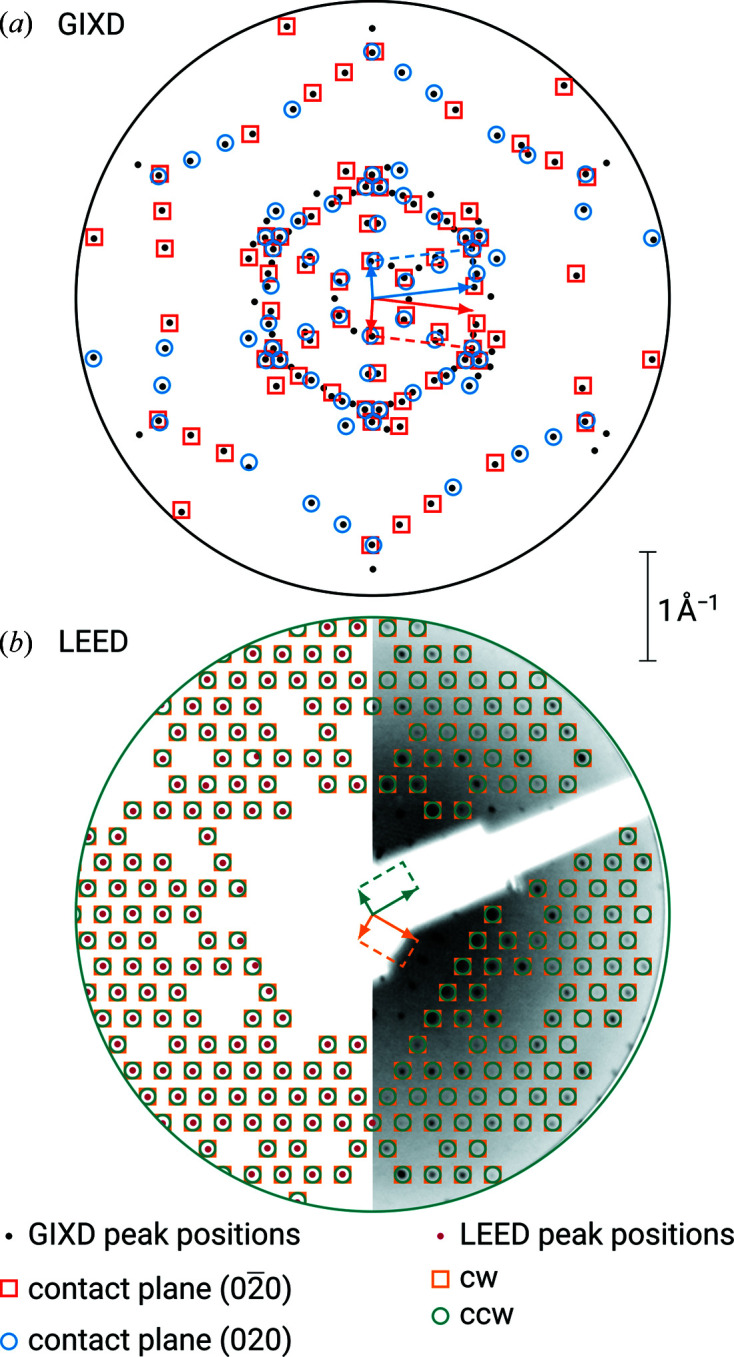
Same as Fig. 2 ▸, but for *trans*-DBPen on Cu(111). The results of the indexing of the oriented crystals are given with respect to the 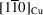 direction. As opposed to the GIXD data (*a*), any reflection in the LEED image (*b*) can be explained by either group of mirror-symmetric unit cells (*i.e.* either cw or ccw) due to the commensurate epitaxial relation.

In Fig. 6 ▸, the (*q_x_*, *q_y_*) positions of the extracted diffraction peaks and the corresponding calculated values from the indexing result are shown for GIXD and LEED. A clear difference in the two diffraction patterns can be observed.

Our results show that the dimensions in the monolayer are significantly larger (see Tables 1 ▸ and 3 ▸).

## 5. Summary and discussion

For all studied molecules, which exhibit various orientations (*i.e*. possess various contact planes), the developed mathematical framework to extract the parameters of the surface unit cells from the underlying three-dimensional lattices could be confirmed experimentally: the parameters of the two-dimensional unit cells calculated from previously obtained three-dimensional data (Tables 3, 4 and 5 in Simbrunner *et al.*, 2022 ▸) correlate with the results of indexing the *x* and *y* components of the reciprocal-lattice vectors (Tables 1 ▸, 3 ▸ and 4 ▸ here). Thus, in a next step, a direct comparison with data obtained from primarily two-dimensional diffraction methods (*i.e*. LEED) is possible. This is advantageous for the analysis of epitaxial differences in the monolayer and in the multilayer.

All errors given in the result tables are exclusively numerical errors (standard deviations) obtained from our algorithm (Simbrunner *et al.*, 2021*b* ▸). They do by no means reflect the absolute uncertainties, which are larger due to the occurrence of systematic and unsystematic errors that are not known precisely. In terms of LEED, *e.g.*, systematic errors stem from uncertainties in the calibration of the device and the determination of the experimental peak positions in the LEED images. A detailed error analysis can be found in the work of Sojka *et al.* (2013*a* ▸).

In Table 1 ▸, the parameters of the two-dimensional unit cells obtained in our GIXD and LEED experiments on the four molecules are separately listed for the two groups of mirror-symmetric cells. Mean values and standard deviations of all parameter sets are calculated over all azimuthal orientations of each unit cell. It can be observed that the uncertainties of the lattice parameters *a*′, *b*′ and γ′ in the GIXD experiments are in the range of 0.5 to 3‰ (on average about 1.5‰). In the LEED experiments, the numerical uncertainties of the lattice parameters *a*′, *b*′ and γ′ are on average about 0.2‰ in the commensurate systems and 1‰ in P2O/Ag(111).

Yet, it is the very nature of things that restrictions apply to the analytic method in two dimensions. For *trans*-DBPen/Cu(111) with the orientation (0±20), two sides (*a* and *c*) of the three-dimensional unit cell can be directly determined. For PTCDA/Ag(111) and P2O/Ag(111) at least one side (*a*′ = *b*) is accessible. In contrast, for lattices with orientations where all Miller indices are non-zero, as in the case of DCV4T-Et2/Ag(111), no vector of the two-dimensional lattice is directly accessible from the three-dimensional lattice. There is, however, access to three diagonals of different planes of the three-dimensional unit cell or one of its supercells (see Table 4 ▸). In PTCDA/Ag(111) *b*′ represents the shorter, and in P2O/Ag(111) *b*′ represents the longer diagonal of the rhomboid which is spanned by the vectors **a** and **c**. In Table 5 ▸, we compare these parameters between the unit cells in the monolayer and in the multilayer.

**Figure 7:**
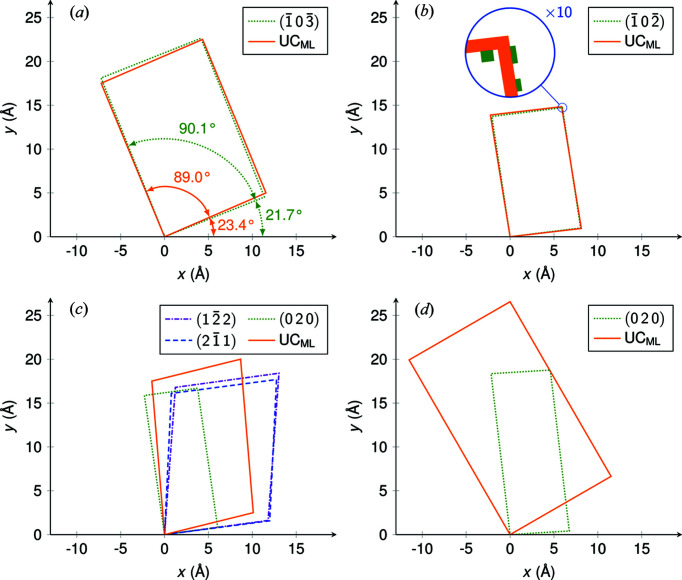
Real-space visualization of the results compiled in Table 1 ▸, (*a*) PTCDA/Ag(111), (*b*) P2O/Ag(111), (*c*) DCV4T-Et2/Ag(111) and (*d*) *trans*-DBPen/Cu(111). The length scales in all four panels are identical, and only the solutions with positive Δϕ are illustrated here for clarity. Broken lines depict the surface unit cells extracted from the GIXD measurements, where (*uvw*) indicate the contact planes of the epitaxially oriented crystals. Solid lines show the surface unit cells of the monolayer (UC_ML_) in contact with the substrate, as measured with LEED. The first substrate lattice vector is oriented parallel to the *x* axis. For PTCDA/Ag(111) in panel (*a*) the angles γ′ and Δϕ are explicitly given as a quick reference.

In Fig. 7 ▸, the surface unit cells of our examples in the monolayer and multilayer are visualized for direct comparison. Comparing our results of GIXD and LEED experiments, the following phenomena may be observed:

(i) Variations in the azimuthal alignments with respect to the substrate. For PTCDA/Ag(111) and P2O/Ag(111), the azimuthal alignment in the *xy* plane with respect to the substrate remains relatively constant in the monolayer and in the multilayer. For *trans*-DBPen/Cu(111), however, Δϕ is ±30° in the monolayer and ±3.5° in the multilayer; this implies a strong effect of the substrate acting as a template and therefore preserving the adsorbate’s hexagonal alignment (rotated by an angle of 30°) in the contact layer [see Fig. 7 ▸(*d*)].

(ii) Changes of the cell parameters in the three-dimensional crystal structure (*e.g*. due to strain). In the monolayer, our examples show various epitaxial properties: commensurism and point-on-line coincidences. In the multilayer, the cell parameters can change significantly. In PTCDA/Ag(111), the length of the vector **a**′ (*i.e*. **b** in the three-dimensional lattice) decreases by about 3% in the multilayer [see Fig. 7 ▸(*a*) and Table 5 ▸]. This may be explained by strain (Krause *et al.*, 2002 ▸). The area of the two-dimensional unit cell, however, remains almost constant. In P2O/Ag(111), the surface unit cells in the monolayer and the multilayer are quite similar [see Fig.  ▸7(*b*)]; the area is about 2% larger in the contact layer (see Table 3 ▸).

(iii) Formation of polymorphs in the multilayer. In the case of DCV4T-Et2/Ag(111), in the monolayer the presence of only one unit cell is observed, whereas in the multilayer three polymorphs with various contact planes were detected. The analysis demonstrates the close relationship between these three distinct unit cells in the *xy* plane [see Fig. 7 ▸(*c*)].

(iv) Distinct changes of the unit cells. In the case of *trans*-DBPen/Cu(111), the two-dimensional unit cell in the monolayer is much larger than the corresponding cell in the multilayer [see Fig. 7 ▸(*d*)]. Furthermore, due to the rectangular shape of the unit cell and its unique azimuthal orientations (60° rotational symmetry and Δϕ = 30°), all reflections can be explained by each group of mirror-symmetric unit cells. This also results in a particular commensurate relationship between substrate and adsorbate.

## 6. Conclusion

A comprehensive mathematical framework has been developed to correlate the parameters of the two- and three-dimensional lattices. Knowing the orientation, *i.e*. the Miller indices of the contact plane, and parameters of the three-dimensional unit cell enables the calculation of the parameters of the surface unit cell. This was experimentally verified by indexing only the *x* and *y* components of the reciprocal-space vectors (*q_x_*, *q_y_*) of four example crystalline molecular adlayers from previous GIXD experiments. These results were compared with recent LEED data obtained from the same molecule–substrate combinations, elucidating the properties of the surface unit cell of the contact layer (*i.e*. first monolayer). Our examples give insight into various phenomena of epitaxial growth such as changes of the crystallographic lattice and azimuthal alignment up to the formation of polymorphs.

## Figures and Tables

**Table 1:** Unit-cell parameters *a*′, *b*′ and γ′ for PTCDA/Ag(111), P2O/Ag(111), DCV4T-Et2/Ag(111) and *trans*-DBPen/Cu(111), experimentally obtained from rotated GIXD (using only the *q_x_* and *q_y_* data) and LEED experiments Mean values and standard deviations of all parameter sets are calculated over all azimuthal orientations of each unit cell. (*uvw*) indicate the contact planes of the epitaxially oriented crystals; the epitaxial relationships of the *a* axis of the adsorbate lattices are specified by the angles Δϕ indicating rotation clockwise (−) and counter-clockwise (+) with respect to the [110] axis of Ag(111) and Cu(111), respectively.

GIXD					LEED			
(*uvw*)	*a*′ (Å)	*b*′ (Å)	γ′ (°)	Δϕ (°)	*a*′ (Å)	*b*′ (Å)	γ′ (°)	Δϕ (°)
PTCDA/Ag(111)								
±(103)	12.214 (24)	19.494 (36)	−90.16 (10) [89.84 (10)]†	−21.6 (1)	12.5881 (15)	18.9359 (11)	−89.001 (16)	−23.410 (10)
	12.242 (19)	19.495 (23)	90.08 (5) [−89.92 (5)]†	+21.7 (1)	12.5882 (15)	18.9352 (3)	89.000 (15)	+23.414 (5)
P2O/Ag(111)								
102	8.105 (10)	13.869 (14)	−92.04 (5) [87.96 (5)]†	−7.2 (1)	8.1665 (57)	14.057 (29)	−92.44 (10)	−6.727 (91)
102	8.096 (5)	13.875 (17)	91.51 (14) [−88.49 (14)]†	+7.1 (2)	8.1701 (31)	14.064 (28)	92.490 (38)	+6.670 (80)
DCV4T-Et2/Ag(111)								
122	11.911 (7)	16.827 (4)	−78.03 (9)	−7.7 (1)	10.4125 (21)	17.5672 (22)	−80.819 (12)	−13.895 (6)
211	12.070 (15)	16.133 (12)	−79.70 (3)	−7.6 (1)				
020	6.121 (11)	16.032 (12)	−90.54 (24)	−7.9 (1)				
122	11.909 (6)	16.836 (8)	77.99 (7)	+7.8 (0)	10.4127 (12)	17.5665 (13)	80.819 (11)	+13.898 (3)
211	12.084 (15)	16.125 (24)	79.74 (3)	+7.5 (1)				
020	6.110 (4)	16.034 (17)	90.53 (9)	+7.7 (2)				
*trans*-DBPen/Cu(111)								
020	6.746 (7)	18.497 (24)	−93.48 (4)	−3.5 (1)	13.2815 (1)	23.0021 (23)	∓90.000 (3)	∓30.002 (27)
020	6.759 (6)	18.481 (43)	93.24 (8)	+3.5 (1)				

† 180° symmetry (*cf.* Simbrunner *et al.*, 2022 ▸).

**Table 2:** Elements *M*_11_, *M*_21_, *M*_12_ and *M*_22_ and determinants of the epitaxy matrices of PTCDA/Ag(111), P2O/Ag(111), DCV4T-Et2/Ag(111) and *trans*-DBPen/Cu(111), obtained from the LEED experiments

*M*_11_	*M*_21_	*M*_12_	*M*_22_	Determinant (**M**) = Area_a_/Area_s_
PTCDA/Ag(111)				
4.9999	0.9999	2.0000	6.9990	32.995
P2O/Ag(111)				
3.0000	1.9998	0.3813	5.5512	15.891
DCV4T-Et2/Ag(111)				
3.9999	2.9996	1.0000	6.9999	24.999
*trans*-DBPen/Cu(111)				
6.0000	0.0001	3.0000	9.0003	54.002

**Table 3:** Area of the two-dimensional unit cell for PTCDA/Ag(111), P2O/Ag(111), DCV4T-Et2/Ag(111) and *trans*-DBPen/Cu(111), calculated from the specular scan *q*_spec_ in X-ray diffraction and the volume from GIXD (Simbrunner *et al.*, 2020 ▸, 2021*a* ▸), compared with the area obtained from GIXD and LEED experiments

Molecule/substrate	Miller indices (*uvw*)	*q*_spec_ (Å^−1^)	Vol. (Å^3^)	Area calculated† (Å^2^)	Area GIXD (Å^2^)	Area LEED (Å^2^)
PTCDA/Ag(111)	±(103)	1.947 (2)	773.0 (28)	239.5 (9)	238.1 (6)	238.32 (2)
P2O/Ag(111)	±(102)	1.942 (2)	363.5 (4)	112.3 (2)	112.3 (1)	114.74 (24)
DCV4T-Et2/Ag(111)	±(122)	1.857 (2)	662.5 (14)	195.8 (5)	196.1 (2)	180.57 (2)
	±(211)	1.828 (2)	661.1 (36)	192.3 (11)	191.7 (1)	
	±(020)	1.828 (2)	673.5 (13)	98.0 (2)‡	98.0 (2)	
*trans*-DBPen/Cu(111)	±(020)	1.660 (2)	944.8 (13)	124.8 (2)‡	124.6 (3)	305.62 (2)

† *cf.* Equation (6).

‡ gcd = 2.

**Table 4:** Correlations between the diagonals in the three-dimensional lattice and the parameters of the two-dimensional unit cell for the ±(122) and ±(211) orientations in DCV4T-Et2/Ag(111) The predicted numbers (from the three-dimensional unit cell) and the determined and calculated numbers from the two-dimensional data sets in GIXD and LEED are itemized. The respective uncertainties are given in brackets.

Diagonal	2D lattice	Predicted	GIXD	LEED
±(122)				
diag(2**a**,**b**) short	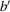	16.849 (32) Å	16.832 (8) Å	17.567 (2) Å
diag(2**a**,**c**) long	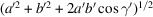	22.563 (38) Å	22.549 (21) Å	21.803 (8) Å
diag(**b**,**c**) short	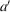	11.907 (11) Å	11.910 (6) Å	10.413 (2) Å
±(211)				
diag(**a**,2**b**) short	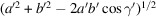	18.326 (84) Å	18.343 (31) Å	18.938 (9) Å
diag(**a**,2**c**) long	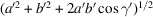	21.773 (77) Å	21.807 (28) Å	21.803 (8) Å
diag(**b**,**c**) short	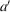	12.062 (56) Å	12.077 (16) Å	10.413 (2) Å

**Table 5:** Ratios of epitaxially comparable parameters between the multilayer (numerator) and the monolayer (denominator) in PTCDA/Ag(111), P2O/Ag(111), DCV4T-Et2/Ag(111) and *trans*-DBPen/Cu(111) The respective calculated propagated uncertainties are given in brackets.

Molecule/substrate	PTCDA/Ag(111)	P2O/Ag(111)	DCV4T-Et2/Ag(111)			*trans*-DBPen/Cu(111)
Orientation	±(103)	±(102)	±(122)	±(211)	±(020)	±(020)
*a*					0.5873 (9)	0.5084 (7)
*b*	0.9714 (20)	0.9917 (12)				
*c*					0.9127 (8)	0.8038 (15)
diag(*v* **a**,*u* **b**) (short/long)			0.9581 (4) (short)	0.9686 (17) (short)		
diag(*w* **a**,*u* **c**) (short/long)	1.0295 (15) (short)	0.9866 (22) (long)	1.0342 (10) (long)	1.0001 (13) (long)		
diag(**b**,**c**) (short/long)			1.14438 (6) (short)	1.1598 (16) (short)		
